# The Value of Desmethylclozapine and Serum CRP in Clozapine Toxicity: A Case Report

**DOI:** 10.1155/2012/592784

**Published:** 2012-08-15

**Authors:** Khalid Abou Farha, Andre van Vliet, Henderikus Knegtering, Richard Bruggeman

**Affiliations:** ^1^QPS Netherlands B.V. 9713 GZ Groningen, The Netherlands; ^2^PRA International, 9471 GP Zuidlaren, The Netherlands; ^3^Lentis Mental Health Organisation, 9725 AG Groningen, The Netherlands; ^4^Rob Giel Onderzoekscentrum, Department of Psychiatry, University Medical Centre Groningen, University of Groningen, 9700 RB Groningen, The Netherlands

## Abstract

Clozapine, an atypical antipsychotic, has proved to be superior to other antipsychotics in treating patients with refractory schizophrenia. An increased plasma clozapine level above the therapeutic window may be associated with serious adverse events including paralytic ileus. Clozapine toxicity may occur in association with infection or after drug overdose. In a medical emergency situation, differentiating between a toxic clozapine ingestion and an infection-induced toxicity might be hindered by associated CNS changes and by the clozapine modulation of the inflammatory process. This may delay prompt initiation of a tailored treatment strategy. Here, we report a case of paralytic ileus developed within the context of clozapine toxicity. Although the underlying cause of toxicity was not clinically obvious, giving antimicrobial therapy resulted in an improvement in the patient's clinical condition. This report indicates the value of serum levels of C-reactive protein and desmethylclozapine, major metabolite of clozapine, in the treatment of aetiologically unclear clozapine toxicity.

## 1. Introduction

Clozapine (CLZ), an atypical antipsychotic, has proved to be superior to other antipsychotics in treating patients with refractory schizophrenia [1]. Nevertheless, clozapine use might be limited by a number of serious adverse events including paralytic ileus, a CLZ-induced anticholinergic effect [2]. Paralytic ileus associated with clozapine treatment might be infection-induced [3, 4] or due to a toxic clozapine ingestion (overdose). Differential diagnosis can be challenging to the treating physician given the associated CNS changes, such as confusion, sedation delirium or even coma, and by the clozapine modulation of the inflammatory process [5–10] that may mask *clinical signs and laboratory markers* of *inflammation.* In this report, we discuss the value of serum CRP and the CLZ metabolite, desmethylclozapine, in the differential diagnosis and treatment of aetiologically unclear clozapine toxicity.

## 2. Case Presentation

**Figure 1:**
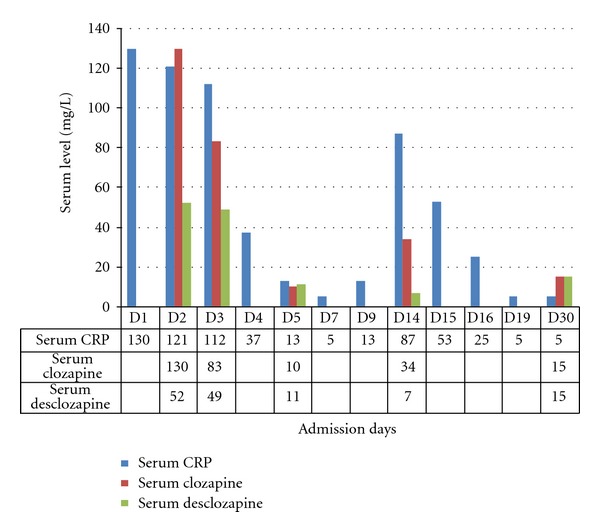
Profile of serum CRP (mg/L), clozapine, and norclozapine (measured in *μ*g/L, changed to mg/L and then *multiplied by 100 for ease* of viewing) levels during admission.

**Figure 2:**
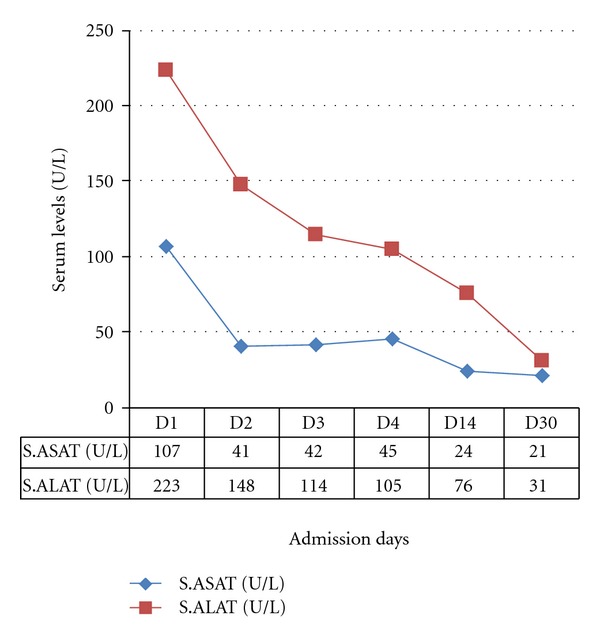
Profile of serum transaminases, ASAT and ALAT during admission.

A schizophrenic patient, male, 44 years old, was referred to the emergency room because of symptoms of dyspnoea, coughing, abdominal pain, nausea, vomiting, diarrhea, and somnolence. The gastrointestinal symptoms began a few days before his admission. The patient is a nonsmoker and has no similar occurrences in his medical history. His outpatient medications included lithium (1000 mg/day), CLZ (300 mg/day), and omeprazole (20 mg/day) for gastrointestinal symptoms. He had been taking CLZ for more than 10 years. The patient was admitted to the gastrointestinal surgery ward. On admission, a physical examination revealed a respiratory rate of 24 breaths/minute, blood pressure 135/100 mmHg, high pulse rate (125 bpm), normal body temperature of 37.1°C, oxygen saturation of 94%, diminished vesicular breath sounds on pulmonary auscultation, abdominal distension, and tenderness, hypertympanic note on abdominal percussion, and hypoactive intestinal sounds. Laboratory tests revealed an elevated CRP (130 mg/L), a normal leucocyte count of 9.5 × 10^9^/L, normal polymorph nuclear neutrophil (PMN) count of 7.5 × 10^9^/L (<80% of total leucocyte count) with no left shift, serum ASAT 107 U/L (reference value < 40 U/L), serum ALAT 223 U/L (reference value < 45 U/L), serum creatinine 173 umol/L (reference range 70–110 umol/L), plasma glucose 14.3 mmol/L (reference range 3.5–7.8 mmol/L), and serum sodium (Na) level of 134 mmol/L (reference range 135–145 mmol/L). A plain X-ray of the abdomen revealed dilated small bowel loops. Nonobstructive dilatation of the small intestine and transverse colon was confirmed on CT scan. A thorax X-ray revealed no pulmonary infiltrate. Determination of the plasma level of the antipsychotics revealed a CLZ level of 1301 *μ*g/L (reference therapeutic range 200–600 ug/L), desmethylclozapine (Norclozapine, NCLZ) level of 515 *μ*g/L making an NCLZ : CLZ ratio of approximately 40%. The lithium level was 0.56 mmol/L (reference therapeutic range 0.6–0.8 mmol/L). A diagnosis of paralytic ileus secondary to clozapine intoxication was made. CLZ and lithium were immediately discontinued. The patient received an i.v. nutritional supplement including NaCL 0.9%, potassium chloride to compensate for the diarrhoea-induced potassium loss and gastrointestinal decompression using nasogastric tube. Because of a suspected pneumonia, amoxicillin/clavulanic acid i.v. at a dose of 200 mg TID was administered for 10 days. Within the first week after institution of therapy and discontinuation of the antipsychotics, there was simultaneous improvement in the patient's condition, return of a normal gastrointestinal function, and a decline in CRP to <5 mg/L and CLZ plasma level to 103 *μ*g/L with an NCLZ level of 110 *μ*g/L making an NCLZ : CLZ ratio of 107%. Given that, lithium (400 mg/day) and CLZ (50 mg/day) were readministered on day 9 postadmission (8th day of amoxicillin/clavulanic acid administration). Under CLZ readministration, the patient developed progressive watery diarrhoea and abdominal pain. Physical examination revealed abdominal tenderness, reduced intestinal peristaltic sounds, normal body temperature of 36.5°C, blood pressure of 120/80 mmHg, and a pulse rate of 104 bpm. Serum levels of liver transaminases were within normal range (ASAT, 24 U/L) or mildly elevated (ALAT, 76 U/L). Similarly, serum alkaline phosphatase (ALP) was not elevated, 77 U/L (reference value <120 U/L). Although the leucocyte count was normal (4.6 × 10E9/L), there was reelevation in CRP (87 mg/L) and an increase in CLZ (336 *μ*g/L) with an NCLZ level of 71 *μ*g/L, making an NCLZ : CLZ ratio of approximately 21%. Faecal culture excluded the presence of pathogenic staphylococci, salmonella enteridis, and clostridia. Given that the onset of diarrhoea was simultaneous with readministration of CLZ, the CLZ was again discontinued on day 13 and a conservative approach adopted until the patient's condition improved 2 days later (i.e., day 15 postadmission). After complete stabilization of the somatic condition and psychiatric profile the patient was discharged with the following medications: risperidone depot 25 mg/2 weeks, lithium 800 mg/day and paroxetine 10 mg/day. Figures 1 and 2 demonstrate the changes in CLZ, NCLZ and CRP levels and serum transaminases during the course of the patient's illness and hospital admission.

## 3. Discussion

In this report, our patient demonstrated, on admission, a toxic high plasma CLZ level accompanied by paralytic ileus and an elevated CRP. There was no conclusive clinical, radiological or laboratory evidence of an infection-induced inflammatory response, such as fever, lung neutrophil infiltration, leucocytosis/neutrophilia, or PMN left shift. Such clinical presentation might lead to uncertainty as to the underlying cause of high CLZ, overdose versus infection-induced, and hence in selecting a treatment strategy including early administration of antimicrobial therapy. Nevertheless, administering antimicrobial, amoxicillin/clavulanic acid, to the patient led to improvement of his clinical condition and was followed by a decline in the CRP level. Typically, fever and neutrophilia are clinical hallmarks of infection. Absence of classic clinical signs of infection may be expected in clozapine toxicity settings. PMNs are first line of defence against bacterial infection. Neutrophils employ an oxidative enzyme system, the myeloperoxidase (MPO) system, to generate potent bactericidal oxidants including superoxide, hydrogen peroxide, and hypochlorous acid [5, 6]. CLZ and its metabolite NCLZ have been reported to exhibit a cytotoxic effect, in a concentration-dependent manner, on the PMNs and their myeloid precursors [6]. In this regard, CLZ and NCLZ can be bioactivated in the peripheral blood PMNs by the MPO system to reactive nitrenium ions which are cytotoxic to PMNs. This toxic effect causes acceleration of the PMN physiologic cell death cycle [6, 7]. Also, clozapine in a concentration-dependent manner has been reported to reduce cold, drugs, and Lipopolysaccharides- (LPS-) induced-body hyperthermia [8–10]. The CLZ body temperature reducing effect is mediated through its 5 HT 1A agonistic and 5 HT 2A antagonistic effect which in turn decreases heat production and increase heat loss through cutaneous vasodilatation.

The major metabolite of clozapine in serum is desmethylclozapine. Demethylation of clozapine is mediated mainly by CYP 1A2 (70%) and to a lesser extent by CYP 3A4 [3, 4, 11, 12]. Under normal conditions, serum levels of NCLZ and CLZ are correlated. An NCLZ : CLZ ratio of >77% that increases with treatment duration has been reported in the literature [4, 11, 13]. Approximately 2–5 hours after clozapine administration, concentrations of NCLZ may exceed those of clozapine [13]. Earlier reports indicated an association between infection and CLZ toxicity [3, 4]. The presumed mechanism for this association suggests that infection downregulates CYP 1A2 by about 90% through increase in circulating IL-6, interferon, and TNF-*α* [3, 4]. This, in turn, interferes with the normal CYP 1A2 metabolic conversion of CLZ and hence the ratio of NCLZ : CLZ may decrease. The patient in this report has been using omeprazole as a concomitant medication for gastrointestinal complaints. Omeprazole is a CYP 1A2 and CYP 3A4 inducer that has been reported to reduce CLZ plasma level by 41.9%–44.7% [11]. The observed elevation of CLZ in this patient would, therefore, confirm inhibition of the CLZ metabolizing enzymes and clarify the observed low NCLZ : CLZ ratio (40%). Meanwhile, *infection-induced increases in IL-6* production stimulate hepatocytes to synthesize and secrete acute-phase proteins such as CRP [3]. Taken together, the final result will be a high (toxic) plasma clozapine level, decrease in NCLZ : CLZ ratio, and elevated serum CRP level. Recognition of these laboratory findings could be of crucial importance in psychiatric emergency settings. Finding high toxic clozapine concentration, low NCLZ : CLZ ratio and elevated CRP level, even in the absence of recognizable clinical signs of infection, can be used as an indirect indication of infection-induced clozapine toxicity and hence justify the early use of antimicrobial therapy.

Another interesting observation is the association between diarrhoea and increased CRP together with decline in the NCLZ : CLZ ratio to 21% after having reached 107% on the 5th admission day, see Figure 1. The diarrhoea started on the 8th day of amoxicillin/clavulanic acid administration. This may suggest an antibiotic associated diarrhoea (AAD). However, the route of amoxicillin/clavulanic acid administration (i.v.) makes an infectious origin of AAD unlikely and the negative result from the faecal culture excluded diarrhoea of infectious origin. Similarly, an antibiotic-induced noninflammatory disturbance in the function of the normal intestinal flora, leading to accumulation of high-molecular-weight carbohydrate in the colon and, therefore, causing osmotic diarrhea [14] cannot explain the elevated CRP and decline in normal CLZ : CLZ ratio. Also, normal serum levels of ASAT (half-life time 12–24 hour) and ALP would argue against recent liver cell injury as a cause of diarrhoea. The observed mild elevation in serum ALAT (<2 times ULN) could be explained by the observed high s. ALAT (223 U/L) at admission and the relatively long half-life time of circulatory ALAT (37–57 hour) that resulted in slow decline in serum ALAT concentration. A more conceivable explanation is the early administration of clozapine to a not completely healthy intestinal epithelium. The patient has been under treatment with omeprazole for GIT complaints. Paralytic ileus is associated with bowel compression, which, in turn, leads to irritation and inflammation of the intestinal epithelium [15], and CLZ has been reported to induce colitis and diarrhoea [16]. Taken together, readministration of clozapine to the patient under such conditions might have increased the inflammatory state of the intestinal epithelium leading to diarrhoea and increased secretion of intestinal cytokines including IL-6 [17, 18] with elevated CRP and decreased NCLZ : CLZ ratio.

In conclusion, we suggest that an elevated CRP and a reduced NCLZ : CLZ ratio might help in the differential diagnosis between an overdose and an infection/inflammation induced clozapine toxicity and, therefore, help prompt selection of treatment strategy. Further investigations are warranted to confirm this suggestion.

## References

[1] (2005). Management of clozapine-induced side effects. *Graylands Hospital Drug Bulletin*.

[2] Pegah Pajouhi P, Bourgeois JA (2007). Clozapine, fluoxetine, and benztropine associated ileus: case report. *Jefferson Journal of Psychiatry*.

[3] Darling P, Huthwaite MA (2011). Infection-associated clozapine toxicity. *Clinical Schizophrenia & Related Psychoses*.

[4] Wetherby E Clozapine serum concentration and infectious processes. http://www.bcmhas.ca/NR/rdonlyres/5893C8EB-5022-456D-AF1A-5C110343D708/37163/BCMHAS_Psychopharmacology_Newsletter_Clozapine.pdf.

[5] Bonvillain RW, Painter RG, Ledet EM, Wang G (2011). Comparisons of resistance of CF and Non-CF pathogens to hydrogen peroxide and hypochlorous acid oxidants in vitro. *BMC Microbiology*.

[6] Williams DP, Pirmohamed M, Naisbitt DJ, Maggs JL, Park BK (1997). Neutrophil cytotoxicity of the chemically reactive metabolite(s) of clozapine: possible role in agranulocytosis. *Journal of Pharmacology and Experimental Therapeutics*.

[7] Robinson DS (2006). Clozapine agranulocytosis: mechanism of drug toxicity. *Primary Psychiatry*.

[8] Blessing WW (2004). Clozapine and olanzapine, but not haloperidol, reverse cold-induced and lipopolysaccharide-induced cutaneous vasoconstriction. *Psychopharmacology*.

[9] Blessing WW, Seaman B, Pedersen NP, Ootsuka Y (2003). Clozapine reverses hyperthermia and sympathetically mediated cutaneous vasoconstriction induced by 3,4-methylenedioxymethamphetamine (ecstasy) in rabbits and rats. *Journal of Neuroscience*.

[10] Ootsuka Y, Blessing WW (2003). 5-Hydroxytryptamine 1A receptors inhibit cold-induced sympathetically mediated cutaneous vasoconstriction in rabbits. *Journal of Physiology*.

[11] Frick A, Kopitz J, Bergemann N (2003). Omeprazole reduces clozapine plasma concentrations: a case report. *Pharmacopsychiatry*.

[12] Linnet K, Olesen OV (1997). Metabolism of clozapine by cDNA-expressed human cytochrome P450 enzymes. *Drug Metabolism and Disposition*.

[13] Weigmann H, Härtter S, Fischer V, Dahmen N, Hiemke C (1999). Distribution of clozapine and desmethylclozapine between blood and brain in rats. *European Neuropsychopharmacology*.

[14] Hogenaur C, Hammer HF, Krejes GJ, Reisinger EC (1998). Mechanism and management of antibiotic-associated diarrhea. *Clinical Infectious Diseases*.

[15] Mukherjee S Ileus. http://emedicine.medscape.com/article/178948-overview.

[16] Pelizza L, Melegari M (2007). Clozapine-induced microscopic colitis: a case report and review of the literature. *Journal of Clinical Psychopharmacology*.

[17] Vitkus SJD, Hanifan A, McGEE DW (1998). Factors affecting Cocoa-2 intestinal epithelial cell interleukin-6 secretion. *In Vitro Cellular & Developmental Biology*.

[18] Reisinger EC, Fritzsche C, Krause R, Krejs GJ (2005). Diarrhea caused by primarily non-gastrointestinal infections. *Nature Clinical Practice Gastroenterology and Hepatology*.

